# Business in Dental Practice

**Published:** 1906-01-15

**Authors:** George F. Burke

**Affiliations:** Detroit, Mich.


### BUSINESS IN DENTAL PRACTICE.

BY GEORGE F. BURKE, D.D.S., DETROIT, MICH.

Read before the Detroit Dental Society, October 12, 1905.

When business is mentioned in this connection it should not be inferred that the commercial element should predominate over the professional. There is a great difference between an objectionable commercialism in the practice of Dentistry, and such a conduct of our affairs as shall result in a financial balance on the right side of the ledger at the end of each year. When we consider that it is this balance, and its wise investment which determines the extent of our future estate, this subject becomes worthy of our serious attention.

When many of us look back over the period during which we were starting our practices, we realize very cleary that it would have been to great advantage to us, if we had served apprenticeships with well established practitioners of the right type.

Experience is a mighty teacher and these older men would have given us much valuable advice. Had we observed closely we might have seen wherein lay their strength, as well as their weaknesses.

These older dentists have learned by experience that all or some things taught in dental colleges and published in dental journals do not always come to pass. They have learned much of the human side of life, and they have discovered that there is much hypocrisy. They have seen patients foresake dentists of ability for those whose knowledge of conservative dentistry could be held on a needle point. They have learned not to believe everything they hear and not to miscontrue what they see. We younger men could have learned much of true value by associating ourselves with these older men, providing they conducted their practices along progressive lines.

One of the essential business propositions that first confronts us is exactness in recording our business transactions. We have ready made systems of bookkeeping, but few master any of them.

Relative to our bookkeeping, a great many in the profession adopt no system and their methods are very lax. This feature in our practice is worthy of much more attention than is usually paid to it. The card index system is in many ways very desirable, and is used by many at the present time, and there are many excellent ledgers on the market.

The matter of keeping records is of great importance. It protects the operator from imposition on the part of some patients concerning the permanency of work that has been done for them, and one can also judge much of the value of various forms of work that has been standing in the mouths of patients for a long time.

One of the greatest evils in dental practice is that of long credits, and yet with traditions and customs as they are to-day, it is frequently necessary to extend credit to most patients. As it is awkward and embarrassing to ask for a settlement after each sitting the extension of credit cannot be avoided. However, long credits are exasperating in practice and a dentist should so conduct his practice that it will be apparent to his patients that he is opposed to unlimited credit.

There are two important points in connection with the subject of credit—To whom shall we extend credit, and how much time can we afford to give those to whom we deem it advisable to give it.

As many patients who apply to a dentist for treatment are totally unknown to him, especially in the larger cities, it becomes necessary for one to acquire some information in regard to their manner of paying other accounts. A very desirable way in which to acquire such information is through reports from a commercial credit company. The best time to secure such a report is after they have applied for an examination, and before they have secured their first treatment. While such reports are not always reliable, still they are more so than any we can secure on our own responsibility, and may do away with a great deal of annoyance in the future.

If a report on a patient indicates that he is extremely slow or does not pay at all, it is well to ask for a settlement at the end of each sitting and if he refuses to do this, let him go elsewhere. If a patient has the reputation of being moderately slow in paying accounts, we should have an understanding with such a person that his account is to be paid for within a certain period.

There are a great many poor collectors in our profession, which works an injustice to ourselves, to our families, as well as to those to whom credit is extended. If patients go along indefinitely without paying their bills, it ultimately results in the dentist sacrificing some of his dignity. The longer an account runs, the more difficult it becomes to collect, the interval of time seems to lessen the obligation of the debtor. After a patient has owed a bill for a long time, it very often happens that he will seek out another dentist, when he needs such service, and pay him if he is a good collector.

Except where the dentist has an understanding to the contrary with individuals, he should send out his accounts on the first day of each month. After a patient has received a statement the third or fourth time, it is a good plan to send a well-worded and polite letter calling attention to the same. If then there is no satisfactory explanation of the delay, there should be no let up on such a person until a settlement is brought about in some way. A capable and tactful collector will often render good service by applying to such a patient in person.

A professional man should never be indifferent to the condition of a poor unfortunate individual, or one who has met with reverses in pursuing the collection of his accounts. While it is well to be prompt in our dealings, we should also extend charity where such is clearly indicated.

Associated with the close collection of accounts should go the prompt payment of obligations. Carelessness in this respect is a great draw-back to any one, and in our profession, where the invested capital is small and the obligation not necessarily large, it is inexcusable not to form the habit of paying legitimate accounts promptly. It is usually true that dentists are poor not of necessity, but because their business methods are lax, and because they allow themselves to drift into shiftless habits.

Many in our profession show poor judgment in the purchase of their instruments, and very often one finds many instruments in an office of no practical use. As the years go by there is bound to be a great deal of useless material accumulate. This seems inevitable. It would be well for the most of us to see a practical test of every instrument that we contemplate buying, and at the same time study it carefully.

It is not meant that one should be niggardly about his equipment; on the other hand a well appointed office inspires respect among patients, and gives the operator much confidence and satisfaction.

Relative to the payment for our supplies, while there are some practitioners who are prompt, there is too much carelessness shown by many in this particular. Comparatively few take advantage of the 10 percent discount offered by the dental depots, in paying for one hundred dollars worth of supplies in advance. This represents quite a saving, and it would be a very difficult matter for any of us to invest our money to better advantage. It is a very desirable thing to do and with responsible houses, it is perfectly safe. When favors are shown in the courtesies of commercial relationship, the dentist who discounts his bills is quite certain to receive such attention.

An operator's fees should be based on the amount of time and skill required in the performance of his work, and must also depend in a great degree on the customs of the locality in which he lives. If a practitioner's fees are lower than his neighbors, there may be a suspicion that he is not so capable. But if a man can give real high grade service, it is uncommon for him to lose patronage because of high fees. The fact of maintaining good fees tends to establish confidence in a man and draws to him a more desirable class of patients. But when a young man starts in practice in a large city, he cannot reasonably expect to get as high fees as some of the well established practitioners. When he gradually gains a good patronage, he should carefully raise his fees so far as his ability and environment will justify.

The investing of our savings is a very important consideration, and should be studied with great care. Our professional usefulness does not continue unabated until old age, and unless we save something during our prosperous period, we may come to want in our older years. Doubtless many of us have been told that it creates a much more comfortable feeling to be, when we are old, in a position to dispense of a little charity instead of being compelled to receive it.

As to the variety of investments suitable for a professional man to make, this is a question that depends on location and conditions. It is an excellent plan to remain away from the stock exchange and grain markets, also avoid the purchase of such stock as gold mines, oil wells and coffee plantations. We would be much better off if we contented ourselves with a more reasonable rate of interest than many of these concerns promise. Investments of a speculative nature are trying on the nerves, and tend to divert our attention from our professional duties.

First mortgages, improved real estate, good bonds and safe dividend paying stock are some of the more desirable investsments for us to make. It is an interesting thing to watch how interest will accumulate as the years roll by. A few hundred dollars saved and invested well, during the most prosperous year's of one's career, will give a reasonable competence. The old men in the profession, who are independent for the most part, are those who have saved something from each year's income and invested it safely, rather than those who have speculated.

While the practice of dentistry will yield a satisfactory income for those who are capable and who apply themselves diligently, still one's annual stipend is not so large that he can afford to look lightly upon the business side of his career.

The various dental faculties would do their graduating students a marked kindness, if they were to include in their curriculum a thoroughly complete and instructive course of lectures upon this subject.

If a man fulfills the obligations of good citizenship, develops his professional attainments to their highest state of perfection, and provides for those dependent upon him by a strict adherance to a sound financial policy, it can be said of him that his life has been well spent.

### DISCUSSION.

Dr. G. B. Watkins: I thoroughly believe in a young dentist starting out with an old practitioner, if he can get with the right kind. The experience would be worth an untold amount in many ways, and from a financial point would pay well, as most of us need money very badly when starting out to practice.

I thoroughly believe in good bookkeeping, not like one of our oldest practitioners of several years ago. I asked him what his practice amounted to? Well, he said he did not know, as he did not keep any account of it, and only kept track of what was due him. A dentist *should* keep a full account of his business and a record of every filling and every other operation made by him. I have kept records of every operation for twenty-five years and many times they have been of great advantage to me. You will frequently have patients with three, four or more different dentists work in their mouths. If a filling comes out a short time after you have done work for them, they will swear it was your filling. Now don't argue with them but produce your chart and you can convince them in one half a minute whether it was your poor work or the other dentists. If it was the other dentist's filling, they will have much more respect for you in the future.

I thoroughly agree with the essayist in regard to credits, we give entirely too much credit. We should send our bills the first of each month regularly, and not wait more than from thirty to sixty days before sending after it. I have never had any success in writing notes about it. If you have an intelligent office girl, send her and tell her what to say. If they pay at all, she will get it. I have succeeded in this way where professional collectors have failed. We would all like to keep clear of asking for our money, but the time comes when we must have it to meet our own bills. I think a report should be kept in our society of deadbeats and slow payers, a system could be arranged that would be very simple when we could get a report on anyone in about a minute.

The essayist speaks of the prompt payment by the dentist of his obligations. Well, that is a subject that I have had something to do with this past summer. As a whole I must say that I believe our Detroit dentists are as generous a class as can be found anywhere. They paid me more money than I thought it was possible to raise from the profession in this city, and the large majority paid it promptly. But we have a few who hated to pay up until the last minute, a few more who were offended when we asked for it, and still a few who did not pay at all. Those who were hard up we forgave and those who held on to their money from lack of good business principles, we hope will benefit from the paper of the evening. Nothing will help a man's standing more than to pay his debts promptly, and as the essayist states, take advantage of all discounts and lay something away for a rainy day.

Dr. F. W. Holt: The point that we should keep close tract of our business transactions is a most essential factor in successful practice for reasons well stated by the essayist. I use the card index with great satisfaction. I had a case recently which promised to give me not a little trouble, but fortunately I was able to produce a complete record of the transaction on my card. I have had much difficulty in determining the credit system. You can't tell good pay by the manners or clothes of your patients. The smoothest talkers will often turn out the greatest "dead beats." Even people occupying good positions in society are slow to pay their accounts, and many such people live by their wits. Some people who are really honest and mean to pay will spend their money on their vanities and keep their doctor or dentist waiting for his fee. As to fees and furnishings these are pleasant things to think about, but we must use discretion in both cases. Charge all your services are worth and supply yourself with such furnishings as you can afford. But above all keep your office and instruments clean and in condition for the best service. A troublesome business feature to manage is broken appointments. Patients can't understand that it is any great loss to the dentist when they can't keep their appointments and rebel when asked to pay for such lapses. If we expect to make a rule that broken appointments should be paid for, unless twenty-four hours notice is given, we must put it in black and white with the patient at the time the appointment is made. Old patients who know of the rule will not take liberties without notification, but children and strangers should be made to feel the importance of keeping all appointments. I have found it a good practice to send a note to patients at certain intervals that they should come to the office for an inspection of their mouths. There are some patients that will appreciate such attention and be very grateful while others may think that you are trying to make business and resent it. All such misunderstandings may be corrected by a previous conference with the patients and the establishment of a list of patients who wish to be notified. Patients have a habit of coming late to appointments without any good reason or excuse. I make it a practice to deal sternly with such patients. If much late I insist on setting another date and try to have such patients understand that it is not set at their convenience but at such a time as it shall be agreeable to me. Patients will appreciate such treatment and not leave you with idle time which you can't afford to loose.

Dr. F. W. McDonald: In regard to sending notices to patients I should fear that we were in danger of unprofessional conduct. If such a system were generally employed it would not be long until some envious or aggressive person would be tempted to introduce into his notices business advertising of a decidely questionable character. I would suggest that all such notices ought to be written and of a personal character so as to avoid possible offense. They should be worded in dignified and ethical language and sent only to such persons as may have desired them and so arranged for them previously.

Dr. T. J. Collins: I have often wondered how far a professional man was warranted in calling upon legal resources in collecting bills. Such proceedings are public and are usually attended with disagreeable public notice, and sometimes unfriendly newspaper comment. And it has seemed to me better for the profession that we suffer the loss in silence rather than go into court.

Dr. N. S. Hoff: The suggestion that the dentist should compel respect for his office by compelling patients to take such time for service as he chooses to give seems rather severe, and also contrary to the usual attitude of the professional man in any calling. A business man or a mechanic may set his own time for attending to ones business, but professional services require such mutual and confidential relations that it is the rule to make every concession to our patients or clients that is practicable. It would seem to be a better plan to endeavor to educate careless patients to realize that we must have prompt attention to appointments; but we cannot be punctilious for the reason that we cannot always meet our appointments promptly without endangering the service which we are rendering the previous appointment. We should try to meet every appointment as promptly as possible, but we cannot afford to be dogmatic with our patients or we shall offend many times, and may do so in a way that shall not work to our business success.

In regard to proper investments, it does seem that there ought to be some safe investments for the slight surplus earnings of professional men. Life insurance used to be considered good, but it seems now that this field is being manipulated by the frenzied financiers to their own enrichment instead of the good and security of the investors. Our saving banks are also too often under suspicion, and the poor professional man has to buy dry oil wells and barren gold mines when he almost knows his money will never return to him again. Perhaps the best thing to do is to get a home or place our money in municipal bonds which are safe, even though they earn small interest.

Dr. G. P. Wood: If the essayist practices what he preaches, I am confident he will succeed. I do not believe it is best to assume an austere or even a dignified attitude with all of our patients. Our patients will respect us, it is true, for exact and punctual business methods; at the same time they will admire and even love us for the many considerations which it is our opportunity to bestow upon them. We can be gracious and still be exact. One of the most successful practitioners of this city lacked the polish of the society gentleman, but he excelled in skill, and built his business on this one acquirement. If he had been more kind, how much greater his success would have been I am not prepared to state, but I am sure his success would not have been less marked. We should not exact from our patient's obligations which we are not prepared to return in kind. If we charge our patients for lost time we should pay them for loss of their time when we keep them waiting. I see no impropriety in asking a deposit from a stranger. An honest patient cannot object, and it would be good evidence that a patient did not expect to pay who would become offended by such a request. In regard to prompt collections I most cordially agree with all that has been said in favor of such a proposition. I keep a list of slow pays on a card conveniently by me, and never lose a chance to remind such people by phone or letter, or by a professional collector as a last resort. I pay my accounts promptly and find that it has a good influence on me reflexly; it makes me work harder to make the money and to collect it promptly after I have earned it.

DR. J. M. THOMPSON: Some dentists can utilize a dignified or austere presence to good effect with tardy or careless patients, but even they can't influence all people. Children cannot be handled nearly so well as by persuasive methods. We can compel respect and compliance to our wishes by the superiority of the service we render our patients. Make every patient believe that you are giving him an especial service. A patient said to me this morning: "If you make my teeth any cleaner I shall think I did not wash my face."

DR. BURKE, in closing the discussion: I do not think it advisable to organize the profession into a commercial agency. There are organizations in every city that will furnish reliable reports for a fee of about fifteen dollars per year. It is not necessary to trace people's dealings with other dentists to find out whether they can be trusted. A person who attempts to beat the dentist has already had experiences with the grocer, butcher and landlord, and his record is on file with these agencies. I had to decline a large fee a little while ago, because the commercial agency gave me an unfavorable report on the patient. I found this particular patient owed the dry goods store, milliners and every one else that she ever had dealings with. I would rather take my chances on a credit company report than from an individual, as personal indorsements many times carry no responsibility, or if they do, we do not care to enforce them, as they may be profitable patients who would thereby become offended. It will be much better for us not to work at all than to give our time and expensive materials to persons who never intend to pay. We can use the time more profitable in reading or seeking recrative exercises.

